# Promoter competition and Polycomb response elements govern transvection efficiency between co-regulated *engrailed* and *invected* genes in *Drosophila*

**DOI:** 10.1093/genetics/iyaf276

**Published:** 2025-12-30

**Authors:** Yuzhong Cheng, Adrienne T Perkins, Fountane W Chan, Natalie D Gehred, Jack R Bateman, Judith A Kassis

**Affiliations:** Eunice Kennedy Shriver National Institute of Child Health and Human Development, Bethesda, MD 20892, USA; Eunice Kennedy Shriver National Institute of Child Health and Human Development, Bethesda, MD 20892, USA; Eunice Kennedy Shriver National Institute of Child Health and Human Development, Bethesda, MD 20892, USA; Eunice Kennedy Shriver National Institute of Child Health and Human Development, Bethesda, MD 20892, USA; Biology Department, Bowdoin College, Brunswick, ME 04011, USA; Eunice Kennedy Shriver National Institute of Child Health and Human Development, Bethesda, MD 20892, USA

**Keywords:** transvection, promoter competition, Polycomb response element (PRE), shared enhancers

## Abstract

Transvection is a phenomenon where gene regulatory elements interact between different chromosomes, adding an additional layer of regulatory control beyond traditional *cis*-interactions. Although transvection effects have been characterized for many individual genes in *Drosophila*, it remains unclear how *trans*-interactions occur among multiple co-regulated genes where enhancers are shared. Here we demonstrate that transvection is supported at the *engrailed-invected* (*en-inv*) locus, where transcription of the two developmental genes is coordinated by common enhancers. Our data show that the presence of the *en* promoter in *cis* to the enhancers prevents *trans*-activation of *inv*, but removal of this promoter enables robust transvection, demonstrating competition between heterologous promoters in *trans*. We also find that local Polycomb response elements (PREs) enhance transvection reliability but are not strictly required for *trans*-activation. Furthermore, our analysis reveals that transvection at this locus is developmentally regulated, occurring efficiently in third instar larval tissues and late-stage embryos but not in early embryonic stages. Finally, we show that *en-inv* transvection can be reconstituted using transgenic constructs at an ectopic chromosomal location, where it produces a striking reciprocal expression pattern between *en* in *cis* and *inv* in *trans*, suggesting that these enhancers can choose to activate one promoter or the other in a stochastic manner.

## Teaser

Transvection (or *trans-activation*) is a process whereby an enhancer on one chromosome activates the promoter of a gene on a paired homologous chromosome. Many duplicated developmental genes in *Drosophila* are co-activated by shared enhancers in cis, which can be facilitated by interactions between promoter-proximal elements. Could co-activation also occur in *trans* when the two promoters are on paired homologs? Contrary to Cheng et al.’s expectations, the *engrailed* enhancers that normally activate two promoters in cis could act in *trans* only when a *cis*-promoter was removed. Furthermore, promoter proximal elements facilitate expression in *trans*, but are not strictly required for *trans*-activation.

## Introduction

The spatial organization of the genome has emerged as a fundamental mechanism governing gene expression in eukaryotes. Chromosomes adopt hierarchical three-dimensional conformations that bring regulatory elements and target promoters into precise spatial relationships while simultaneously partitioning the genome into active and inactive compartments, enabling transcriptional programs that define cell identity (reviewed by Acemel and Lupiáñez 2023; Hehmeyer et al. 2023). Understanding how three-dimensional genome structure influences gene regulation is important for deciphering normal development, disease pathogenesis, and the potential for novel therapeutic interventions.

Many layers of 3D genome organization appear conserved across Eukarya, but the proteins that establish these conformations can differ from species to species. Common to diverse organisms are topologically associated domais (TADs), chromosomal segments of high self-interaction that tend to be flanked by insulator sequences (reviewed by Bhattacharya et al. 2024). In mammalian systems, a model has emerged where TADs are formed by cohesin-dependent loop extrusion, which can be halted by CTCF proteins bound to insulators, thus forming TAD boundaries. While the *Drosophila* genome is also characterized by partitioning into TADs, cohesin and CTCF do not play a major role in TAD formation in this species (reviewed by Matthews and White 2019). Rather, other mechanisms appear to drive local compartmentalization of the *Drosophila* genome, including preferential clustering of similarly modified chromatin states and/or the binding activities of species-specific architectural proteins (Ulianov et al. 2016; Rowley et al. 2017; Bhattacharya et al. 2024).

In addition to the local chromatin folding that creates TADs, eukaryotic genomes also exhibit long-range interactions between chromosomally distal loci that play important roles in coordinating gene expression across multiple genomic regions. In *Drosophila*, Polycomb response elements (PREs) and their associated Polycomb Group complexes are found at many distally-interacting loci (Li et al. 2015; Ogiyama et al. 2018; Brown et al. 2024; Gurgo et al. 2024), and transgenic insertions of PRE sequences can initiate ectopic long-distance interactions (Bantignies et al. 2003; Grimaud et al. 2006; Vazquez et al. 2006; Kwon et al. 2009). Additionally, several studies have identified Tethering Elements (TEs) that mediate long-range interactions between distant genomic loci (reviewed by Li and Levine 2024). Recent analyses have shown that a significant subset of TEs in *Drosophila* overlap with known PREs (Batut et al. 2022; Levo et al. 2022), highlighting that some regulatory elements may serve dual functions in both Polycomb-mediated silencing and broader chromosomal organization.

Adding to the complexity of genome organization in *Drosophila* and other Dipterans is the phenomenon of somatic homolog pairing, where homologous chromosomes are intimately paired from end to end in somatic cells (reviewed by Joyce et al. 2016; Peterson et al. 2021). The close juxtaposition of homologs can permit regulatory DNA to communicate in *trans* between the two alleles of a gene, a phenomenon known as transvection (reviewed by Duncan 2002; Kennison and Southworth 2002; Galouzis and Prud’homme 2021a). In many classical examples of transvection, a mutant allele lacking essential enhancer sequences can be complemented by pairing with a second mutant allele that retains the enhancers but carries mutations in the promoter or coding region, effectively sharing regulatory elements across the paired chromosomes to restore gene function. Several studies have also uncovered important roles for transvection underlying developmental events in otherwise wild-type genetic backgrounds (Johnston and Desplan 2014; Galouzis and Prud’homme 2021b; Antel et al. 2022), emphasizing the importance of understanding mechanisms governing *trans*-gene regulation in this system. However, most classical studies of transvection have focused on individual genes in isolation, leaving open the question of how long-range chromatin interactions are coordinated simultaneously across both *cis* and *trans* configurations, particularly for co-regulated genes that must respond to common developmental or environmental cues.

**Fig. 1.**
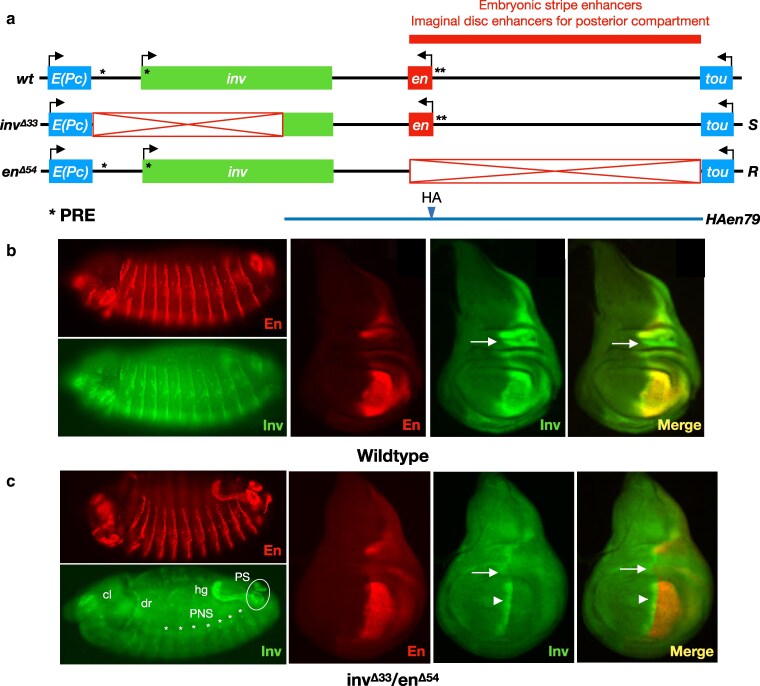
Trans-activation of *inv* is not supported in the presence of the *en* promoter in *cis*. a) schematic of the *en*-*inv* locus. The region of the red bar contains enhancer DNA required for embryonic stripes and the imaginal disk posterior compartments. Structures of wild type, *inv^Δ33^*, and *en^Δ54^* alleles are shown (red boxes, deleted regions; asterisks, PREs; “*S*,” “Sender” chromosome carrying intact enhancers for the imaginal disk posterior compartment and embryonic stripes but lacking the *inv* promoter; “*R*,” “Receiver” carrying an intact *inv* promoter but lacking the enhancers carried on the *S* chromosome. Line below the genomic locus shows the extent of DNA in the transgene *HAen79* with an HA tag encoded on the N-terminus of the En protein. b) wild-type pattern of En and Inv staining in embryos (left) and wing imaginal disks (right). Embryo is stage 13, anterior left, dorsal up, lateral view. c) En and Inv staining in *inv^Δ33^*/*en^Δ54^* embryos (left) and wing disks (right). Inv staining is only observed in regions controlled by enhancers in *cis* on the *R* chromosome (peripheral nervous system (PNS, white asterisk), dorsal ridge (dr), posterior spiracles (PS), hindgut (hg), and clypeolabrum (cl) in embryos, and a stripe at the A/P boundary in disks (arrowhead), and not in regions controlled by enhancers in *trans* from the Sender chromosome. Arrows indicate a region at the wing hinge with higher Inv staining relative to En in wild-type wing disks. Embryo is late stage 13, anterior left, dorsal up, dorsolateral view. At least ten wing disks and stage 13 embryos were examined for each genotype, and a representative disk or embryo is shown.

The Drosophila *engrailed* (*en*) and *invected (inv)* gene complex is one of many developmentally important gene pairs in *Drosophila* (Levo et al. 2022). Like other gene pairs, *en* and *inv* encode highly related proteins that share expression patterns and function (Gustavson et al. 1996). En is required for many aspects of *Drosophila* development, including embryonic segmentation and the formation of the posterior compartment of imaginal disks (reviewed by Joyner et al. 2024), while Inv plays a more supporting role and is not required for viability in the laboratory (Gustavson et al. 1996). Both *en* and *inv* are contained within a 113-kb domain between the genes *Enhancer of Polycomb* (*E(Pc)* and *toutatis* (*tou*)), two genes with unrelated expression patterns and functions (Fig. 1a; De et al. 2020; Jenkins et al. 2022). Transcription of *inv-en* is controlled by a large number of enhancers distributed throughout the *inv-en* domain, many of which are located upstream of the *en* transcription unit (Cheng et al. 2014), and by the Polycomb-group of transcriptional repressors (Moazed and O'Farrell 1992). There are four PREs in the *inv-en* domain, two at *inv* and two at *en* (Cunningham et al. 2010; Brown and Kassis 2013). When *inv* and *en* are not expressed, the PREs at both genes interact and the nucleosomes within the domain are modified with the repressive Polycomb chromatin mark, H3K27me3 (Brown et al. 2024). When the two genes are transcribed, part of the domain is modified with the active chromatin mark H3K27ac and the promoter-proximal PREs interact, suggesting they serve as TEs to coordinate the transcription of these two genes (Brown et al. 2024).

Knowing that somatic chromosomes are paired in *Drosophila*, we sought to test whether enhancers that regulate *en* and *inv* when they are on the same chromosome (in *cis*) could also regulate these two genes when they are on homologous chromosomes (in *trans*). Our data show that enhancers upstream of the *en* transcription unit can act in *trans*, but only when the *en cis*-promoter is removed, suggesting a *cis*-preference that was not anticipated. Furthermore, we showed that the PRE/TEs facilitate but are not required for *trans*-activation, consistent with experiments that show these elements coordinate but are not required for *cis*-activation of paired genes (Levo et al. 2022). Finally, we demonstrated transvection between two transgenes, one carrying *en* and one carrying *inv*, which suggests the enhancers often activate only one promoter or the other, something not seen at the endogenous locus.

## Materials and methods

### Deletions in the endogenous *inv-en* domain

All new deletions were made using CRISPR/Cas9 following the procedures in https://flycrispr.org as described in Cheng et al. (2023). The coordinates for the deletions are listed in Table 1.

**Table 1.** *inv-en* mutants and transgenes used in this paper.

Name	Deleted sequences	Reference
*inv^Δ33^*	11466259..11499376	(De et al. 2019)
*inv^Δ45^*	11469702..11514649	(Cheng et al. 2014)
*inv^Δ62^*	11466241..11528210	this study
*inv^Δ64^*	11466241..11529821	this study
*en^Δ47^*	11529803..11576474	this study
*en^Δ48^*	11528279..11576472	this study
*en^Δ54^*	11522241..11576472	this study
*en^Δ110^*	11466238..11577472	(De et al. 2019)
*en^E^*	11496174..11537511	(Gustavson et al. 1996)

Large transgenes	DNA Coordinates^a^	Reference
*HAen45*	11516495..11561426	(Cheng et al. 2014)
*HAen79*	11499333..11578495	(Cheng et al. 2014)
*HAinv84*	11438746..11522240	(Cheng et al. 2014)

^a^All coordinates are on chromosome 2R, genome v6.

### Genetic crosses and immunostaining

For all crosses except *inv^Δ33,^* which is homozygous viable and vigorous, mutant chromosomes were balanced over either *CyO, actin-GFP,* or *CyO, Kr-GFP*. For embryo collections, the *Kr-GFP* chromosome was used because GFP is expressed in early embryos. For larval collections, 4–7 female virgins of one genotype were crossed to males of the other and transferred every 2–3 d. All crosses were kept at 25 °C. Ten to 12 GFP negative 3rd instar larvae were collected (with the exception of *HAen45/en^Δ54^* where 6 larvae were used), dissected, and stained with antibodies as previously described (Cheng et al. 2014). Primary antibodies used were guinea pig anti-Inv (1:5000, Cheng et al. 2014), rabbit anti-EN (1:500, Santa Cruz Biotechnology, Inc), rabbit anti-GFP (1:2000, Invitrogen A11122) or mouse anti-GFP monoclonal (1:250, sc-9996). Alexa Fluor secondary antibodies (Invitrogen) were used: goat-anti-guinea pig 488 at 1:1000; goat-anti-rabbit 555 Superclonal at 1:1000 for larval stainings, 1:500 for embryo stainings; and Donkey-anti-mouse 488 at 1:500. Embryos and disks were mounted in Vectashield with DAPI (Vector Labs). For embryos, overnight collections (0–16 h) and daytime collections (0–7 h) were done, and hundreds of embryos (stages 1 to 16) were immunostained and examined.

### ChIP-seq

ChIP was performed on larval brains and disks as previously described (Langlais et al. 2012). Two biological samples were taken for each genotype. ChIP-seq libraries were made and analyzed as described in De et al. (2019).

## Results

To address whether transvection is supported at the *en-inv* locus, our general strategy was to create flies where *inv* transcription could only occur if enhancers act in *trans*. To accomplish this, we used CRISPR/Cas9 genome editing to engineer “Sender” (*S*) chromosomes carrying various deletions of the *inv* region, leaving intact the enhancers upstream of *en* that control *en-inv* expression in embryonic stripes and the posterior compartment of imaginal disks (Fig. 1a) (Gustavson et al. 1996; Cheng et al. 2014). We further engineered “Receiver” (*R*) chromosomes that delete the enhancer region upstream of *en* but leave intact the *inv* transcription unit. In *trans*-heterozygous flies carrying *R/S* chromosomes, transcription of *inv* in embryonic stripes and imaginal disk posterior compartments is only possible if the enhancers from the *S* chromosome activate the *inv* promoter on the *R* chromosome in *trans*. In some experiments, deletions on the *S* and *R* chromosomes each remove the *en* gene, which is required for embryonic development. In those cases, we employed a transgene, *HAen79*, that carries an HA-tagged functional copy of *en* that is inserted at the attP40 landing site on chromosome arm 2L (Fig. 1a). The 79-kb transgene includes the *en* transcription unit and regulatory DNA and supports development in the absence of the *inv-en* domain (Cheng et al. 2014, 2023; De et al. 2019) but should not participate in *trans*-interactions at the endogenous *en-inv* locus due to its distal location.

### The upstream *en* enhancers show *cis*-preference for the *en* promoter

As a first test, we used *inv^Δ33^*, an *S* chromosome carrying a 33 kb deletion including the *inv* promoter and much of the transcription unit, and *en^Δ54^*, an *R* chromosome that deletes the *en* transcription unit and the upstream enhancers (Fig. 1a, see Table 1 for the coordinates of all deletions and transgenes). Note that *inv^Δ33^* is homozygous viable and fertile, while *en^Δ54^* is homozygous lethal as it lacks the *en* transcript and regulatory DNA. We crossed flies carrying these chromosomes to create *en^Δ54^*/*inv^Δ33^ trans*-heterozygotes and assessed Inv expression in stage 13 embryos and in third instar larval wing disks. In control wild-type organisms, both En and Inv are observed in epithelial stripes in the embryo and in the posterior compartment of the wing disk, where they are largely co-expressed except in the hinge region of the wing disk (arrow in Fig. 1b). In *en^Δ54^*/*inv^Δ33^ trans*-heterozygotes, En staining matches the pattern seen in wild type due to the intact *en* gene on the *S* chromosome (Fig. 1b). However, we see no evidence of Inv staining in embryonic stripes or in the posterior compartment of the wing (Fig. 1c). Rather, Inv staining is restricted to regions controlled by enhancers present on the *en^Δ54^* chromosome, including the clypeolabrum (cl), dorsal ridge (dr), hindgut (hg), posterior spiracles (PS), and cells in the peripheral nervous system (PNS) in embryos and a stripe at the boundary of the anterior (A) and posterior (P) compartments in wing disks (arrowhead points to it in Fig. 1c). Thus, *trans*-activation of *inv* is not supported in *en^Δ54^*/*inv^Δ33^* embryos or wing disks.

**Fig. 2.**
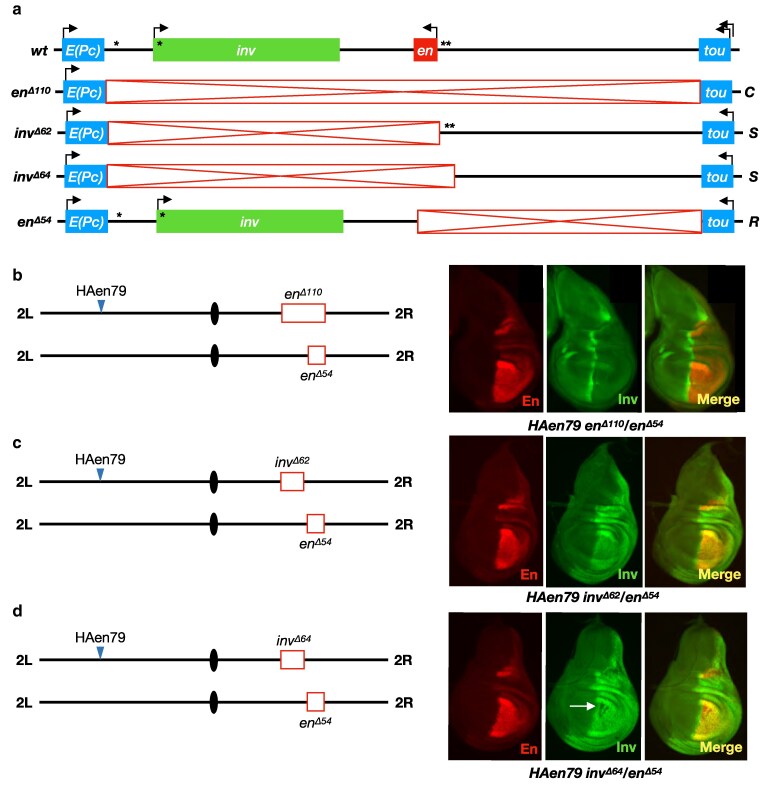
Deletion of the *en* promoter in *cis* permits *trans*-activation of *inv* in the posterior compartment of the wing disk. a) structures of alleles at the *en*-*inv* locus (red boxes, deleted regions; asterisks, PREs; “*C*,” “Control” chromosome; “*S*,” “Sender” chromosome; “*R*,” “Receiver” chromosome). Note that the deletions in *inv^Δ62^* and *inv^Δ64^* each remove the entire *en* transcriptional unit and differ only by 1,611 bp that includes the PREs upstream of *en*. b–d) *trans*-activation of *inv*. In each case, the transgene *HAen79* is present to provide expression of *en* from an ectopic location since each genotype is otherwise embryonic lethal; all En staining therefore results from expression from the transgene, whereas Inv staining results from expression at the endogenous locus. b) control genotype showing no *trans*-activation of *inv*. *en^Δ110^* deletes all enhancers from the chromosome in *trans* to the intact *inv* promoter on the *en^Δ54^* chromosome, resulting in Inv staining only at the A/P boundary of the wing disk (driven by an enhancer in *cis* to *inv* on the *R* chromosome). A representative example is shown from observations of at least 10 disks. c) *trans*-activation of *inv* by the *S* chromosome *inv^Δ62^*. Robust staining of Inv is observed throughout the posterior compartment. d) *trans*-activation of *inv* by the S chromosome *inv^Δ64^*. Inv staining is similar to that observed with *inv^Δ62^* (c), but with patches of variegated expression in some disks (arrow). Three out of nine *HAen79 inv^Δ64^/en^Δ54^* disks examined had patches of no Inv expression (see also Supplementary Fig. 1a). No such patches were observed in ten *HAen79 inv^Δ62^/inv^Δ54^* wing disks.

Previous analyses of transvection have shown that enhancers tend to show a preference for promoters in *cis* relative to promoters in *trans*, and that the presence of a *cis*-promoter can interfere with an enhancer's capacity to support transvection (Geyer et al. 1990, Martínez-Laborda et al. 1992). Although the *inv* promoter is deleted from the *inv^Δ33^ S* chromosome, the *en* promoter remains intact. To address whether the upstream enhancers may be restricted from acting in *trans* by the presence of the *en* promoter, we created a new *S* chromosome, *inv^Δ62^*, that removes the *en* promoter and transcriptional unit in addition to those of *inv* (Fig. 2a). As a control chromosome (*C*), we used *en^Δ110^*, which lacks the *en-inv* domain and therefore cannot act as either Sender or Receiver in transvection. In control *HAen79 en^Δ110^/en^Δ54^* wing disks, En staining shows its expected pattern in the posterior compartment due to the presence of the *HAen79* rescue construct, whereas Inv staining is entirely absent from the posterior compartment (Fig. 2b) but is expressed at a stripe at the A/P boundary as expected. In contrast, *HAen79 inv^Δ62^/en^Δ54^ trans*-heterozygotes show strong Inv expression in the posterior compartment, indicative of robust *trans*-activation of *inv* on the *en^Δ54^ R* chromosome by the enhancers on the *inv^Δ62^ S* chromosome (Fig. 2c).

### Transvection at the *en-inv* locus does not require *en* PREs or *zeste*

Notably, the deletions on the *R* and *S* chromosomes *inv^Δ62^* and *en^Δ54^* are overlapping, indicating that there is no requirement for homology across the 113 kb *en-inv* locus in order to support transvection. However, the two chromosomes carry four well-characterized PREs; two on the *R* chromosome, with one 6 kb upstream of and one within the *inv* promoter, and two on the *S* chromosome, both upstream of the *en* promoter, that may facilitate enhancer-promoter *trans*-interactions through the ability of these PRE sequences to interact with each other (Levo et al. 2022; Brown et al. 2024). We therefore created a new *S* chromosome, *inv^Δ64^*, which is analogous to *inv^Δ62^* but extends an additional 1.6 kb to remove the *en* PREs. In trans-heterozygous *HAen79 inv^Δ64^/en^Δ54^* wing disks, we continue to see robust *trans*-activation of *inv* expression in the posterior compartment, indicating that *trans* PRE-PRE interactions are not necessary for enhancer action in *trans* at this locus (Fig. 2d). However, 3 of 9 *HAen79 inv^Δ64^/en^Δ54^* wing disks had large groups of cells that lacked Inv (Fig. 2d, white arrow), and these patches of cells differed in each disk (Supplementary Fig. 1a). No such patches were seen in ten *HAen79 inv^Δ62^/en^Δ54^* disks. We suggest that the lack of the *en* PREs decreases the ability of the enhancers to act in *trans*, creating a variegated pattern of expression.

Our analyses thus far have focused on the third instar larval wing disk, but the wild type pattern of larval *en* and *inv* expression includes the posterior compartments of other disk tissues. To assess whether *inv-en* enhancer action in *trans* is supported across different tissues, we also examined leg disks for Inv protein in *R/S trans*-heterozygotes. Control *HAen79 en^Δ110^/en^Δ^*^54^ leg disks show En staining in the posterior compartment due to the presence of the *HAen79* construct, while Inv is seen as a discontinuous stripe at the A/P boundary (Supplementary Fig. 2a). However, as we had observed in the wing disk, *HAen79, inv^Δ62^/en^Δ54^* and *HAen79, inv^Δ64^/en^Δ54^ trans*-heterozygotes (with and without *en* PREs on the *S* chromosome, respectively) show staining of Inv throughout the leg disk posterior compartment (Supplementary Fig. 2a), indicating that transvection at the *en-inv* locus is broadly supported across larval tissues.

Prior analyses of transvection have shown that *trans*-interactions at some loci, but not others, are modified by mutations in *zeste* (*z*) (Duncan 2002; Kennison and Southworth 2002). To assess a potential role for *z* in transvection at the *en-inv* locus, we tested two classes of *z* alleles; *z^1^*, a neomorphic allele that modifies expression of the *white* gene in a pairing-dependent fashion, and *z^a^*, a loss-of-function mutation. In either *z^1^* or *z^a^* mutants, we see no obvious change in Inv staining in *HAen79, inv^Δ62^/en^Δ54^* wing or leg disks relative to staining in a wild-type *z* background (Supplementary Fig. 2b), indicating that *z* is unlikely to play a significant role in transvection at this locus.

### *en* and *inv* genes in *trans* to the upstream enhancers compete for activity

**Fig. 3.**
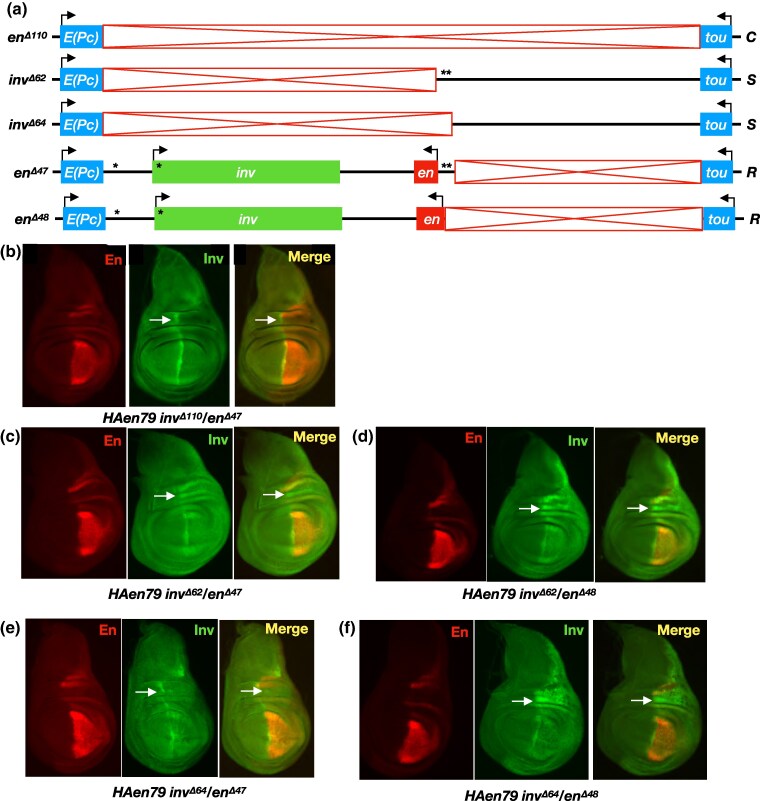
*Trans*-activation of *inv* is compromised by the presence of the *en* promoter on an *R* chromosome. a) Structures of alleles. *C* and *S* chromosomes are identical to those shown in Fig. 2. The *R* chromosomes *en^Δ47^* and *en^Δ48^* include both the *inv* and *en* transcriptional units but differ in the presence or absence of the *en* PREs. b) control genotype *HAen79 en^Δ110^/en^Δ47^* shows no *trans*-activation of *inv* from the *C* chromosome. c) *HAen79 inv^Δ62^/en^Δ47^ trans*-heterozygotes produce Inv staining in the posterior compartment that is less robust relative to Fig. 2 where the *R* chromosome lacks the *en* transcriptional unit. d) *trans*-activation of *inv* in *HAen79 inv^Δ62^/en^Δ48^* wing disks is greater relative to panel (c). e and f), *HAen79 inv^Δ64^/en^Δ47^* and *HAen79 inv^Δ64^/en^Δ48^* disks show patterns of Inv staining that are weaker and often variegated relative to comparable genotypes with the sender chromosome *inv^Δ62^*. Arrows point to the hinge region with high Inv expression in the wild type. At least ten wing disks were examined for each genotype, and a representative disk is shown. *HAen79 inv^Δ62^/en^Δ54^* (Fig. 2c) and *HAen79 inv^Δ62^/en^Δ47^* (Fig. 3c) immunostainings were performed and imaged at the same time. The same was true for the *HAen79 inv^Δ64^/en^Δ54^* (Fig. 2d) and *HAen79 inv^Δ64^/en^Δ47^* (Fig. 3e) immunostainings; though non-quantitative, this difference in intensity between the stainings was well controlled and confirmed by two observers.

Thus far, our data indicate that the presence of the *en* gene in *cis* to the upstream enhancers on the *S* chromosome can prevent *trans*-activation of *inv* on the *R* chromosome. To further explore a potential role for competition between *en* and *inv*, we created a new *R* chromosome, *en^Δ47^*, that contains an intact *en* transcriptional unit in addition to *inv* but lacks the upstream enhancers (Fig. 3a). In combination with an *S* chromosome, *en^Δ47^* provides two potential target promoters in *trans* to the upstream enhancers, *en* and *inv*. In control *HAen79 en^Δ110^/en^Δ47^* wing disks (enhancers deleted from both chromosomes), Inv staining is absent from the posterior compartment as expected, but present in a stripe at the A/P boundary (Fig. 3b). In *HAen79 inv^Δ62^/en^Δ47^ trans*-heterozygotes, Inv staining can be seen in the posterior compartment; however, the staining is reduced relative to *R/S* genotypes that contain a deletion of the *en* promoter and gene body on the *R* chromosome (compare to Fig. 2, c and d). In *HAen79 inv^Δ64^/en^Δ47^* disks, where the *en* PREs are deleted from the *S* chromosome, Inv staining is sparse in the posterior compartment and occurs only in small patches of cells (Fig. 3e). In Fig. 3, an arrow points to the hinge region of the disk, where Inv is the predominant protein, even in wild-type disks (see Fig. 1b). When *en^Δ47^* is the *R* chromosome, Inv is present in this region when the PREs are present on the *S* chromosome (Fig. 3c) and largely absent when they are not (Fig. 3e). Thus, in this case, the PREs on the *S* chromosome are crucial for robust transvection.

We also tested the role of the *en* PREs on the *R* chromosome using the allele *en^Δ48^*, which is analogous to *en^Δ47^* with an additional 1.5 kb deleted that includes the *en* PREs. Both *HAen79 inv^Δ62^/en^Δ48^* and *HAen79, inv^Δ64^/en^Δ48^ trans*-heterozygotes support *trans*-activation of *inv* in the posterior compartment (Fig. 3, d and f), with staining levels apparently stronger than those observed for the analogous *R* chromosome *en^Δ47^* with intact *en* PREs (Fig. 3, c and e). Thus, removing the *en* PREs from the *R* chromosome improved its ability to respond to the enhancers on the *S* chromosome. We hypothesize that when the *en* PREs are in *cis* to the *inv* PREs, these PREs interact and interfere with the trans-activation of *inv*.

### *Trans*-activation of *en* and *inv* is supported during embryogenesis

**Fig. 4.**
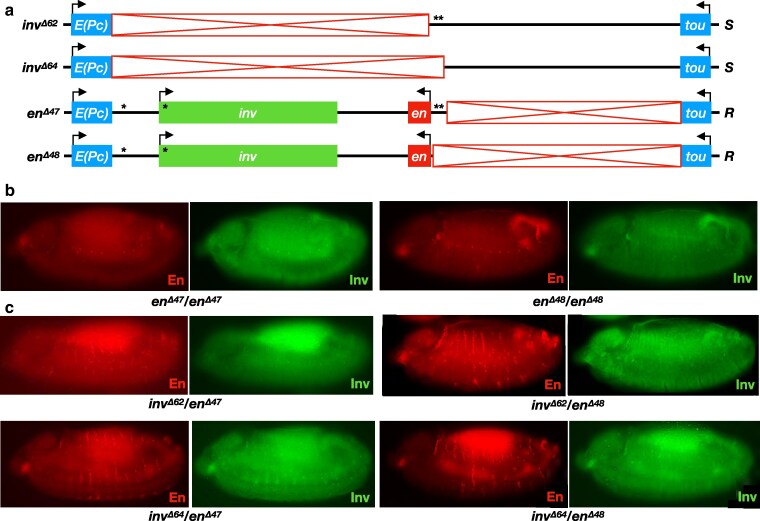
*Trans*-activation of *en* is supported in epidermal stripes of stage 13 embryos. a) structures of alleles. b) Embryos homozygous for *R* chromosome *en^Δ47^* or *en^Δ48^* show no staining of En or Inv in epidermal stripes (note that the *HAen79* transgene is not carried by any embryos shown here; all *en* expression is from the endogenous locus). c) Variegated staining of En is observed in stripes of *inv^Δ62^*/*en^Δ47^*, *inv^Δ64^*/*en^Δ47^*, *inv^Δ62^*/*en^Δ48^*, and *inv^Δ64^*/*en^Δ48^* embryos, with increased staining in genotypes with *en^Δ48^* relative to *en^Δ47^*. Inv staining is generally undetectable in each genotype. All embryos are anterior left, dorsal up, lateral view, stage 13. At least 10 mutant embryos at stage 13 were examined for each genotype, and a representative embryo is shown.

Thus far, our experiments have focused on the third instar larval stage and have therefore relied on the presence of the *HAen79* transgenic construct to rescue the lethality of *en* deletions at the endogenous *en-inv* locus, precluding us from further assessing *trans*-activation of the *en* gene itself. To further explore transvection of *en*, we turned to embryos. While embryos that lack both *en* and *inv* exhibit defects in embryonic segmentation early, *en^Δ47^* and *en^Δ48^* have an intact *en* transcription unit, encode a functional En protein, and embryos that are homozygous for these alleles develop normally through at least stage 13. We suspect this is due to the presence of early stripe enhancers within the *en* transcription unit that provide a small but undetectable amount of En protein early in development; most of the stripe enhancers are located upstream of the *en* PREs (Cheng et al. 2014). No En or Inv stripes were observed in homozygous *en^Δ47^* or *en^Δ48^* embryos at any stage (stage 13 embryos are shown in Fig. 4b), although each protein is found in other tissues for which enhancers are intact on these chromosomes (e.g. PNS, hindgut). In *R/S* allelic combinations between *R* chromosomes *en^Δ47^* and *en^Δ48^* and the *S* chromosome *inv^Δ62^*, En protein is observed in many cells of the epidermal stripes beginning at stage 13 (Fig. 4c), indicating that *en* transcription can be activated by the stripe enhancers in *trans* (Fig. 4c). In contrast, no Inv staining is apparent in the epidermis of either *en^Δ47^*/*inv^Δ62^* or *en^Δ48^*/*inv^Δ62^* embryos, consistent with our observations in the wing disk that the presence of *en* on the *R* chromosome can reduce the *trans*-activation of *inv* (Fig. 3). Similar results were obtained using the *S* chromosome *inv^Δ64^* which lacks the *en* PREs, further supporting that PRE-PRE interactions are not strictly required for transvection at the *en-inv* locus (Fig. 4c).

**Fig. 5.**
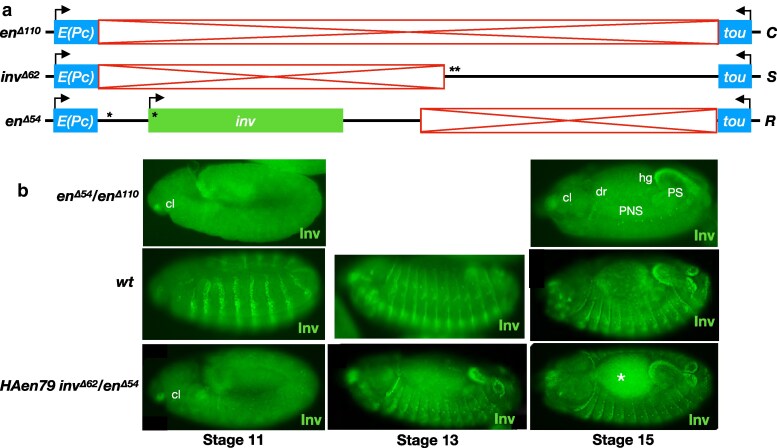
*Trans*-activation of *inv* in epidermal stripes. a) structures of alleles. b) The genotype of the embryos in each row is listed on the left, and the stage of the embryos in each column is listed at the bottom. Top row, In control *en^Δ54^/en^Δ110^* embryos (without the *HAen79* rescue construct), Inv protein is present in tissues controlled by enhancers present on the R chromosome but is absent from the epidermal stripes. Middle row, in wild type embryos (wt), Inv protein is present in the epidermal stripes at all stages shown. Bottom row, *trans*-activation of *inv* in the epidermal stripes of *HAen79 inv^Δ62^/en^Δ54^* is observed at stages 13 and 15 but absent at stage 11. All embryos are anterior left, dorsal up. Asterisk denotes autofluorescence in the yolk. Stage 11 *en^Δ54^/en^Δ110^* and *HAen79 inv^Δ62^/en^Δ54^* embryos are lateral, and *wt* embryos are ventral-lateral. *wt* stage 13 embryo is a lateral view; *HAen79 inv^Δ62^/en^Δ54^* is a dorsolateral view. Stage 15 embryos are dorsolateral views. At least 10 embryos of each stage shown were examined, and a representative embryo is shown.

To confirm that the lack of *inv* transvection in embryonic stripes was due to the presence of *en* on the *R* chromosome, we once again employed *en^Δ54^*, which deletes the upstream enhancers and the *en* transcriptional unit (Fig. 5). In control embryos of *en^Δ54^* in combination with *en^Δ110^* (the deletion of the *en-inv* locus), no staining of Inv is observed in epidermal stripes at any stage (stages 11 and 15 are shown) due to the deletion of the upstream enhancers from both chromosomes. In comparison to wild-type embryos, the *en^Δ54^*/*en^Δ110^* control embryos lacking En on both chromosomes have abnormal development, including no head involution, making late staging hard. We therefore used the *HAen79* rescue construct and assessed *trans*-activation of *inv* in *HAen79 inv^Δ62^/en^Δ54^* embryos that have normal morphology. In *HAen79 inv^Δ62^/en^Δ54^* embryos, Inv staining is present in cells of the epidermal stripes at stages 13 and 15, indicating that *trans*-activation of *inv* is supported by the stripe enhancers on the *S* chromosome. Notably, we do not see evidence of Inv staining in the epidermal stripes of stage 11 embryos, indicating that transvection at the *en-inv* locus is not yet supported at this time of development.

### *Trans*-activation of *inv* is supported between transgenic insertions

**Fig. 6.**
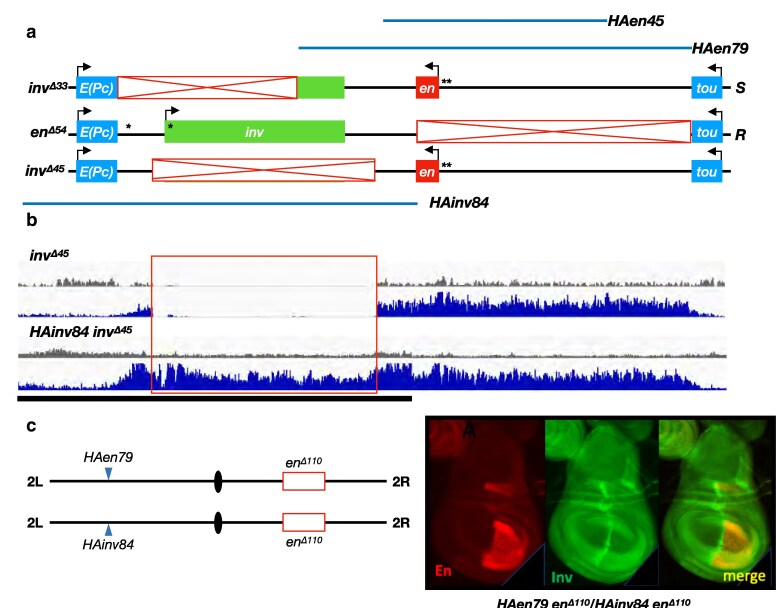
*Transvection studies using large transgenes*. a) schematic of transgenes used. Bars above show the region encoded on Sender transgenic insertions *HAen79* and *HAen45*, whereas the bar below shows the region encoded by Receiver transgene *HAinv84*. Schematics of alleles *inv^Δ33^* and *en^Δ54^* are shown to highlight that the breakpoints of the deletions correspond to the endpoints of the transgenes. *inv^Δ45^* deletes the entire *inv* transcription unit and some flanking DNA. b) ChIP-seq showing the *en-inv* region on *inv^Δ45^* (upper) and *HAinv84 inv^Δ45^* third instar brains and disks, Grey-input DNA, Blue-H3K27me3. Note that the H3K27me3 accumulation in the region of the *inv^Δ45^* deletion (red box) in the *HAinv84 inv^Δ45^* genotype is coming from *HAinv84* transgene. The black line below is the extent of the *HAinv84* transgene; regions flanking the *inv^Δ45^* deletion have higher levels of H3K27me3 because of signal from both the transgene and the endogenous locus. All tracks are 0–5. c) Inv is present at the A/P boundary in *HAen79 en^Δ110^/HAinv84 en^Δ110^* wing disks, showing there is no trans-activation of *inv* in the genotype. A representative example is shown from a total of 10 disks observed.

As a final test of the capacity for the *en-inv* locus to support transvection, we assessed whether transgenes carrying elements of the locus at an ectopic location are sufficient to allow *trans*-activation of *inv* in the posterior compartment of the wing disk using the same logic of “Sender” and “Receiver” elements. The *HAen79* transgene used throughout this study is analogous to the *S* chromosome *inv^Δ33^* in that it contains the complete *en* transcriptional unit and upstream enhancers but lacks the *inv* promoter and part of the *inv* transcription unit (Fig. 6a). In addition, a second Sender transgene, *HAen45* (also inserted at the *attP40* landing site), encodes a truncation of the *HAen79* transgene that includes the complete *en* transcriptional unit but removes regulatory DNA from upstream and downstream enhancer regions (Fig. 6a, Table 1). Finally, the Receiver transgene *HAinv84* is analogous to the *R* chromosome *en^Δ54^*, encoding the complete *inv* promoter and transcriptional unit but lacking the *en* gene and its upstream enhancers (Fig. 6a). Inv expression from the *HAinv84* transgene is similar to that seen from *en^Δ54^* including no stripes in embryos and a stripe at the A/P boundary in disks (Cheng et al. 2014). As stated above, *inv* and *en* are regulated by the Polycomb group genes. In cells where these two genes are not transcribed, the Polycomb repressive chromatin mark H3K27me3 covers the locus from the 3′ end of the *e(Pc)* gene to the end of the 3′ end of the *tou* gene. Likewise, the *HAen79* transgene is also covered by H3K27me3 (De et al. 2019). We wondered whether the *HAinv84* transgene could also form an H3K27me3 domain. To assess this, we performed chromatin-immunoprecipitation followed by next-generation sequencing (ChIP-seq) on third instar larval brains and disks of the genotype *HAinv84@attP40 inv^Δ45^*. The *inv^Δ45^* allele deletes the entire *inv* transcription unit and some regulatory DNA (Fig. 6a), and ChIP-seq shows H3K27me3 over the *inv-en* domain except in the region of the deletion (Fig. 6b). In contrast, H3K27me3 is present throughout the domain in *HAinv84 inv^*∆45*^*larvae, with the middle signal coming from the *HAinv84* transgene. Thus, the *HAinv84* transgene can also form an H3K27me3 domain, likely initiated by the *inv* PREs.

**Fig. 7.**
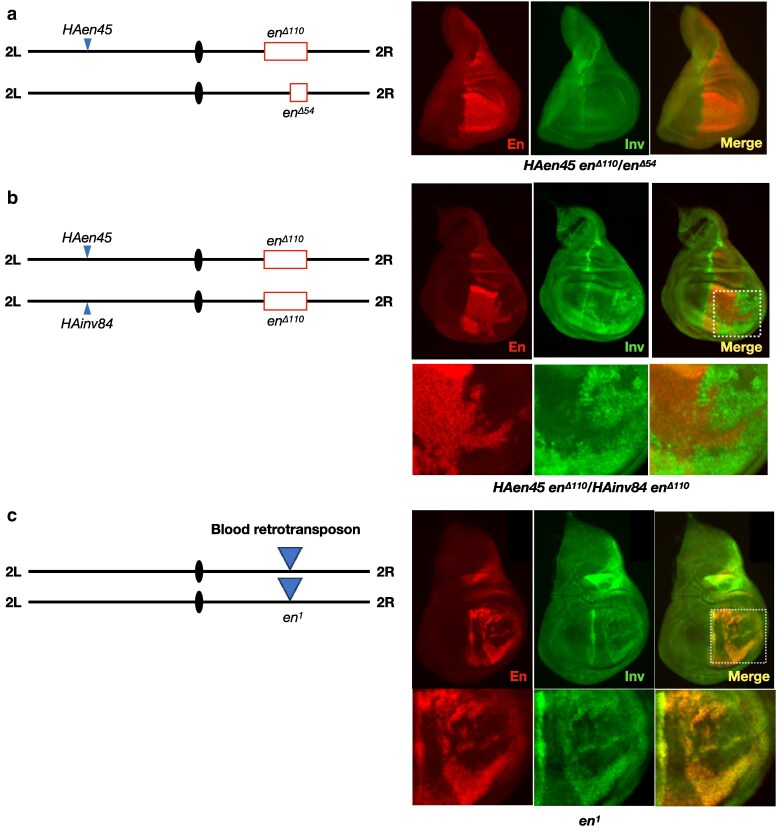
*Trans*-activation of *inv* is supported by the transgene *HAen45*. a) *HAen45* modestly rescues *en^Δ110^/en^Δ54^* embryos that entirely lack *en* at the endogenous locus. Wing disks of this genotype are slightly misshapen, with strong En staining throughout the posterior compartment but Inv staining primarily at the A/P boundary. Six disks were examined, and all had the same pattern of En and Inv expression. b) *trans*-activation of *inv* in *HAen45/HAinv84 trans*-heterozygotes homozygous for *en^Δ110^*. Both En and Inv staining are evident in the posterior compartment, with many cells activating only one gene or the other. The dashed white box highlighted in the merge disk picture surrounds the region of the disk enlarged in the pictures below. Eleven disks were examined, and all had reciprocal patterns of En and Inv expression. c) on the *en^1^* chromosome, a blood retrotransposon (triangle) is inserted approximately 13 kb upstream of the *en* transcription start site (Cheng et al. 2014). En an Inv are variegated and co-expressed in an *en^1^* wing disk. The dashed white box in the merge disk picture surrounds the region of the disk enlarged in the pictures below. Ten disks all showed En-Inv co-expression in this genotype.

We first examined En and Inv staining in *HAen79 en^Δ110^/HAinv84 en^Δ110^*, where 110 kb of the *en-inv* region is deleted from the endogenous locus on both homologs, and the transgenes are therefore the only potential sources of En and Inv proteins. This experiment is analogous to our initial assessment of transvection in *en^Δ54^*/*inv^Δ33^ trans*-heterozygotes, where no transvection was evident; as expected, we once again observed no evidence of *inv trans*-activation in this transgenic combination (Fig. 6c). We then examined the ability of the much smaller *HAen45* transgene to work as a Sender chromosome for transvection. We attempted to assess En and Inv staining in control *HAen45 en^Δ110^/en^Δ110^* wing disks, but this genotype fails to develop sufficient disk tissue due to incomplete rescue from the truncated *HAen45* transgene (Cheng et al. 2014). However, replacing one of the *en^Δ110^* alleles with the smaller *en^Δ54^* deletion (*HAen45 en^Δ110^/en^Δ54^*) produced somewhat deformed but recognizable wing disks where En staining was found throughout the posterior compartment and Inv staining was evident only at the A/P boundary, as expected (Fig. 7a). Finally, we assessed the potential for *trans*-activation of *inv* by the Sender transgene *HAen45* by placing it in *trans* to *HAinv84*, with both inserted at the attP40 site on chromosomal 2L. In this case, *HAen45 en^Δ110^/HAinv84 en^Δ110^* (homozygous for the large *en^Δ110^* deletion) organisms develop into healthy third instar larvae with robust wing disk tissue. In these disks, both En and Inv proteins are observed in the posterior compartment, indicating that transcription of *inv* encoded on the *HAinv84* transgene is activated in *trans* by the enhancers encoded on the *HAen45* transgene (Fig. 7b). Notably, although some cells show evidence of both proteins, in most cells, only En (produced in *cis*) or Inv (produced in *trans*) is seen, resulting in a reciprocal pattern of staining across the wing disk rather than co-expression (Fig. 7b). A similar reciprocal pattern was observed in all 11 disks of this genotype examined, with some variation in the exact staining pattern (see two additional examples in Supplementary Fig. 1b), and therefore appears to be a consistent feature of transvection between the *HAen45* and *HAinv84* transgenes. The reciprocal pattern of expression was surprising to us since En and Inv are usually co-expressed, exist in a gene complex, and are Polycomb-regulated. We wondered whether a reciprocal expression of En and Inv also occurred in a different genetic background. Specifically, in *en^1^* mutants, wing development is disrupted, and staining of wing disks with an antibody that recognized both Inv and En showed variegated expression (Brower 1986), but it was not known whether these two proteins were co-expressed. We therefore stained *en^1^* homozygous larval disks with anti-Inv and anti-En antibodies. Unlike En and Inv in *HAen45 en^Δ110^/HAinv84 en^Δ110^* wing disks, En and Inv proteins are completely co-expressed in *en^1^* wing disks, in variegated patterns that vary in each disk (Fig. 7c, see Supplementary Fig. 3 for more info about *en^1^*). In sum, transgenes carrying sequences from the *en-inv* locus are sufficient to support transvection at an ectopic locus, with enhancer action supported either in *cis* or in *trans*.

## Discussion

**Fig. 8.**
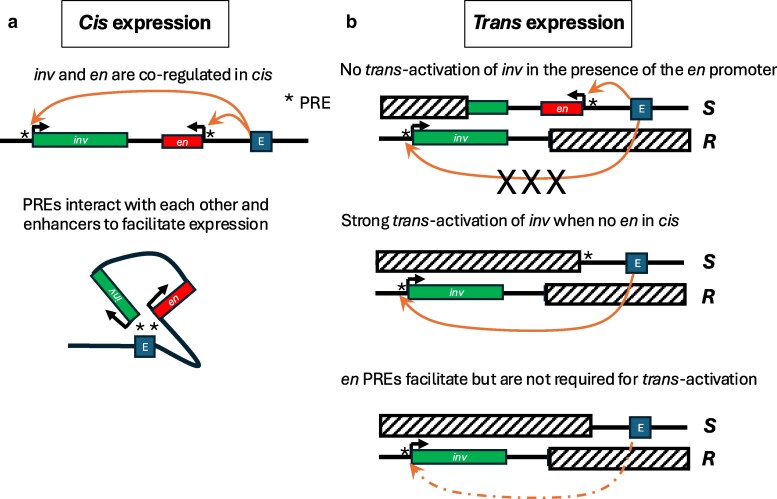
Summary model. a) *cis* expression. The *inv* and *en* transcription units are shown with an arrow showing the start and direction of transcription. The stripe or imaginal disk enhancers are shown by an E in a blue box. In the upper linear model, the enhancers can activate both the *en* and *inv* promoters. In the lower model, the promoter-proximal *en* and *inv* PREs interact with each other and the enhancers to bring the DNAs together and facilitate transcription (Levo et al. 2022; Brown et al. 2024). b) *Trans* expression. The striped boxes indicate DNA deleted from the *inv-en* locus in our transvection assays. The upper panel shows that, in the presence of the *en* transcription unit, the enhancers cannot activate the promoter on the homologous chromosome (indicated by XXX interrupting the arrow). The middle panel shows that when the *en* promoter and transcription unit is removed, the enhancer can now activate the *inv* promoter. In this case, the PREs are present and could act together to facilitate transcription. Bottom panel, when the *en* PREs are not present on the *S* chromosome, *trans*-activation is less robust leading to variegated expression.

Transvection has been well documented at many *Drosophila* loci, but its regulation at complex co-regulated developmental gene loci remains poorly explored. Here, we investigated whether transvection is supported at the *en-inv* locus, where the *en* and *inv* genes are co-regulated by shared upstream enhancers during embryonic segmentation and imaginal disk development (Fig. 8a). The co-expression of these genes is aided by promoter-proximal PREs, which interact with each other and enhancers to facilitate gene expression. Using a CRISPR-based approach to create Sender (*S*) chromosomes containing regulatory DNA, and Receiver chromosomes (*R*) containing the Inv transcription unit, we tested the ability of embryonic stripe enhancers and imaginal disk enhancers to act in *trans* (Fig. 8b). In the presence of the *en* transcription unit on the *S* chromosome, the enhancers could not activate the *inv* promoter (Fig. 8b, top). When the *en* promoter was removed (Fig. 8b, middle), strong *trans*-activation of *inv* occurred. Note that there is no homology between the *S* and *R* chromosomes and that the PREs can facilitate interactions between the two promoters and the enhancer in this configuration. Finally, upon deletion of the *en* PREs (Fig. 8c, bottom), the *trans*-activation is less robust, leading to a variegated pattern of expression.

We were surprised that the *en* upstream enhancers were unable to *trans*-activate *inv* in the presence of an *en* promoter in *cis*. Although preference for a *cis*-promoter has been well documented in transvection at other loci (Martínez-Laborda et al. 1992; Casares et al. 1997; Gohl et al. 2008; Bateman et al. 2012; Mellert and Truman 2012; Lim et al. 2018; Piwko et al. 2019), for the majority of genes tested, weak activation of a promoter in *trans* is still permitted in the presence of a promoter in *cis*. To the best of our knowledge, the *yellow* gene is the only other case that shows a complete lack of observable transvection in the presence of a *cis*-promoter (Geyer et al. 1990; Morris et al. 1999), although transgenic studies have shown this to be position-dependent (Kravchuk et al. 2016). At the *en-inv* locus, the upstream enhancers normally activate both *en* and *inv* promoters simultaneously, and micrococcal nuclease and chromatin capture (micro-C) analysis has shown that the promoter-proximal PREs of *en* and *inv* are physically looped together (Levo et al. 2022; Brown et al. 2024), which likely facilitates long-range clustering of the two promoters with the upstream enhancers. We hypothesized that this PRE-dependent support of long-range promoter clustering would extend to interactions in both *cis* and *trans*, thereby facilitating *trans*-activation of *inv* even in the presence of the *en* promoter, but it must instead be the case that interactions in *cis* are preferred over interactions in *trans*. This *cis*-preference could simply be because the *inv* promoter is too far away to be activated when it is on the homologous chromosome. Note that in our experiments, there is transvection between the *S* and *R* chromosomes that share no homology. It would be interesting to do similar experiments using small deletions or mutations of the *en* and *inv* promoters, leaving the two chromosomes otherwise homologous. In support of a role for PRE-PRE interactions in facilitating transvection, loss of the *en* proximal PREs on the *S* chromosome in *HAen79 inv^Δ64^/en^Δ54^* and *HAen79 inv^Δ64^/en^Δ47^* larvae results in less consistent *trans*-activation of *inv* relative to the genotypes *HAen79 inv^Δ62^/en^Δ54^* and *HAen79 inv^Δ62^/en^Δ47^*, where the *en* PREs are still present on the *S* chromosome. Conversely, when the *en* promoter and its proximal PREs are present on the *R* chromosome in *en^Δ47^*, *trans*-activation of *inv* is reduced relative to the *R* chromosome *en^Δ48^* without the *en* PREs, in this case we hypothesize that the *en* and *inv* PREs in *cis* on the *en^Δ47^ R* chromosome propagate a very stable “OFF” state, while the loss of the *en* PREs on *en^Δ48^* destabilizes this state allowing *trans*-activation of *inv*.

Our analysis of developmental timing of *inv* transvection is consistent with our understanding of the onset of somatic homolog pairing. Maternal and paternal homologs are largely unpaired through the early syncytial divisions, and pairing increases dramatically beginning in cell cycle 14, with many loci plateauing by stage 13–15 (Fung et al. 1998; Gemkow et al. 1998; Child et al. 2021). It is therefore likely that homolog pairing has not progressed sufficiently by stage 11 to support transvection at the *en-inv* locus, whereas *trans*-interactions between homologs have stabilized by stage 13, supporting transvection. Similarly, prior analyses of transvection at other loci have demonstrated transvection in embryos at stage 13 and later (Hendrickson and Sakonju 1995; Sipos et al. 1998; Fujioka et al. 2016), with no evidence of pregastrulation transvection in the absence of the transgenes carrying the gypsy insulator (Lim et al. 2018).

While classic examples of enhancer action in *trans* in *Drosophila* typically involve *trans*-heterozygous allelic combinations of endogenous genes, transgenes that are inserted at common positions on homologous chromosomes can also support this form of transvection. This was first observed using transgenes carrying components of the *yellow* gene (Chen et al. 2002) and later with synthetic fluorescent reporter genes (Bateman et al. 2012; Mellert and Truman 2012). We were surprised to find that the Sender transgene *HAen45* supported *trans*-activation of *inv* on the Receiver transgene *HAinv84* whereas the Sender *HAen79* did not. Both *HAen79* and *HAen45* carry a complete *en* transcriptional unit in *cis* to the upstream enhancers; therefore, we expected both elements to be restricted from acting in *trans*. *HAen79* contains 17 kb more DNA on both the distal and proximal ends relative to the *HAen45* transgene, and we propose that those sequences somehow “lock in” the *cis*-promoter preference of the posterior compartment imaginal disk enhancers.

We were further surprised by the patterns of expression observed for *en* (activated in *cis*) and *inv* (activated in *trans*) in the posterior compartment of *HAen45 en^Δ110^/HAinv84 en^Δ110^* wing disks undergoing transvection, with many cells activating only one or the other gene. Previous analyses of transvection using fluorescent reporters have instead shown that an enhancer will typically co-activate promoters in *cis* and in *trans* in the same cells (Bateman et al. 2012; Mellert and Truman 2012; Lim et al. 2018) with one exception (Kassis 2012 ; Mellert and Truman 2012). Furthermore, the relatively large regions of tissue showing expression of one gene or the other suggest that a decision on which gene to activate (or repress) was made in earlier precursor cells and then maintained in clonal patches as the cells of the disk underwent mitosis. Overexpressing En can lead to its silencing in a variegated manner in wing disks (Guillén et al. 1995; Tabata et al. 1995), producing variegated expression patterns similar to those seen in *en^1^* homozygous disks. In addition, both *HAen79* and *HAen45* can be repressed by En produced by the endogenous *en-inv* domain. This regulation is likely to be direct as En is a repressor, binds to one of the imaginal disk enhancers (IDE), and can repress expression of an IDE-reporter transgene (Cheng et al. 2023). While we have no evidence that En produced from *HAen45* can repress the expression from *HAinv84* (or vice versa), the reciprocal pattern of expression common in *HAen45 en^Δ110^/HAinv84 en^Δ110^* wing disks could, in theory, involve feedback by En and/or Inv proteins, repressing expression of one transgene or the other. To this end, it would be interesting to test whether *en* and *inv* genes carrying loss-of-function point mutations would show a similar pattern of expression in paired transgenes, although the complication of embryonic lethality for *en* loss-of-function mutations would present a challenge.

Three-dimensional analysis of *Drosophila* genome structure using micro-C has identified a class of elements, called tethering elements (TEs), that facilitate interactions between regulatory DNA, including enhancer-promoter and promoter-promoter interactions (reviewed in Li and Levine 2024). In one study (Levo et al. 2022), more than 20 co-regulated developmental gene pairs, including *en-inv*, were identified that contain paired TEs located near their promoters. Removal of a TE from one gene in a pair uncoupled expression of the two genes, and led to stochastic expression of the gene without the TE (Levo et al 2022). The promoter-proximal *en* PRE can function as a TE for the imaginal disk enhancers (Kwon et al 2009), and the PRE at the *inv* promoter can pair with a presumptive enhancer in micro-C experiments (Brown et al. 2024), suggesting it is also a TE. We suggest that many of the TEs identified in gene pairs may facilitate both *cis*- and *trans*-regulatory interactions.

## Supplementary Material

iyaf276_Supplementary_Data

## Data Availability

Fly lines are available upon request. Next-generation sequencing (NGS) data have been deposited in the NCBI GEO database, accession number GSE308802. The authors affirm that all other data necessary for confirming the conclusions of the article are present within the article, figures, and tables. Supplemental material available at GENETICS online.
