# Supplementary material for: Promoter competition and Polycomb response elements govern transvection efficiency between co-regulated engrailed and invected genes in Drosophila
Source: Genetics. 2025 Dec 30;232(3):iyaf276. doi: 10.1093/genetics/iyaf276 (PMC13017635; doi:10.1093/genetics/iyaf276)
Supplement: iyaf276_Supplementary_Data [file iyaf276_supplementary_data.zip › Supplemental Figure legends.docx]

**Supplemental Figure Legends**

**Figure S1. Additional examples of variegated expression of Inv in wing discs.** *A*, two wing discs of each genotype are shown. *A*, *HAen79 inv^∆64^/en^∆54^*.

*B*, *HAen45 en^∆110^/HAinv84 en^∆110^*.

**Figure S2. *Trans*-activation of *inv* is supported in additional larval tissues and is not affected by mutations in *zeste*.** *A*, leg discs with genotypes identical to those shown in Figure 2 show no staining of Inv in the posterior compartment of the leg disc in control *HAen79,* *en^Δ110^/en^Δ54^* discs but robust Inv staining in *HAen79,* *inv^Δ62^/en^Δ54^* and *HAen79,* *inv^Δ64^/en^Δ54^*, with the latter showing higher levels of variegation as observed in the wing disc (Figure 2). *B*, *trans*-activation of *inv* is supported in *HAen79,* *inv^Δ62^/en^Δ54^* carrying the neomorphic allele *z^1^* (top) or loss of function allele *z^a^* (bottom). L-leg disc, W- wing disc, H-haltere disc.

**Fig. S3. En and Inv are co-expressed in *en^1^/en^∆54^*, *en^1^/inv^∆62^*, and *en^1^/en^∆110^* wing discs.** *A*, Mutant chromosomes used in these experiments, same diagrams as in the paper. *B-D*, two wing discs of each genotype showing co-expression of En and Inv. *B*, like in *en^1^* homozygotes, En and Inv expression is variegated in *en^1^/en^∆54^* wing discs. *C* and *D*, expression in *en^1^/inv^∆62^* and *en^1^/en^∆110^* is nearly wildtype. Previous studies have shown that *en^1^* homozygotes have more severe adult wing phenotypes than those of *en^1^* over *en* loss of function and *en-inv* deletion mutants (Kornberg 1981; Eberlein and Russell 1983).

**References**

Kornberg T. 1981. Engrailed: a gene controlling compartment and segment formation in Drosophila. Proc Natl Acad Sci U S A. 78(2):1095-9. <https://doi.org/10.1073/pnas.78.2.1095>.

Eberlein S, Russell MA. 1983. Effects of deficiencies in the engrailed region of Drosophila melanogaster. Dev Biol.100(1):227-37.

<https://doi.org/10.1016/0012-1606(83)90215-4>.
