# Supplementary figures and images for: Promoter competition and Polycomb response elements govern transvection efficiency between co-regulated engrailed and invected genes in Drosophila
Source: Genetics. 2025 Dec 30;232(3):iyaf276. doi: 10.1093/genetics/iyaf276 (PMC13017635; doi:10.1093/genetics/iyaf276)

Supplemental Fig. 1

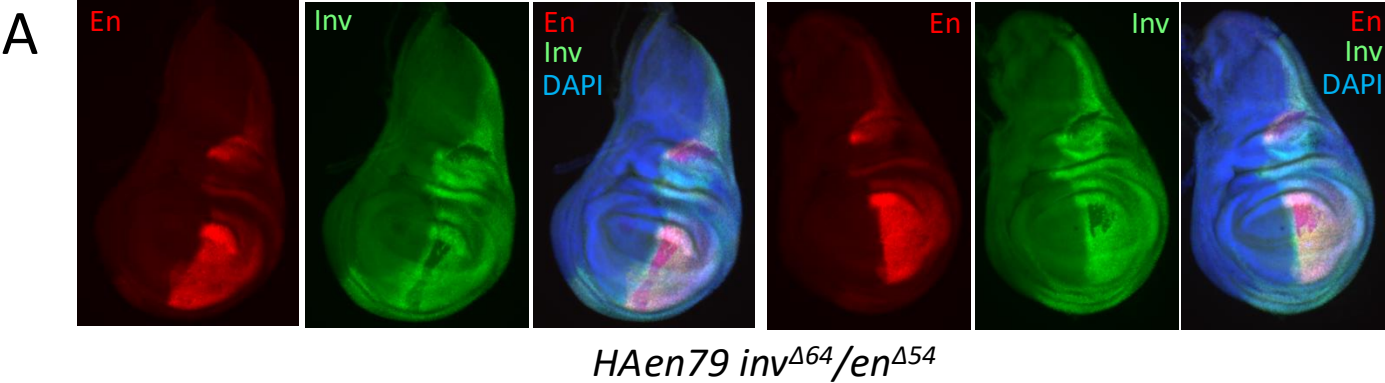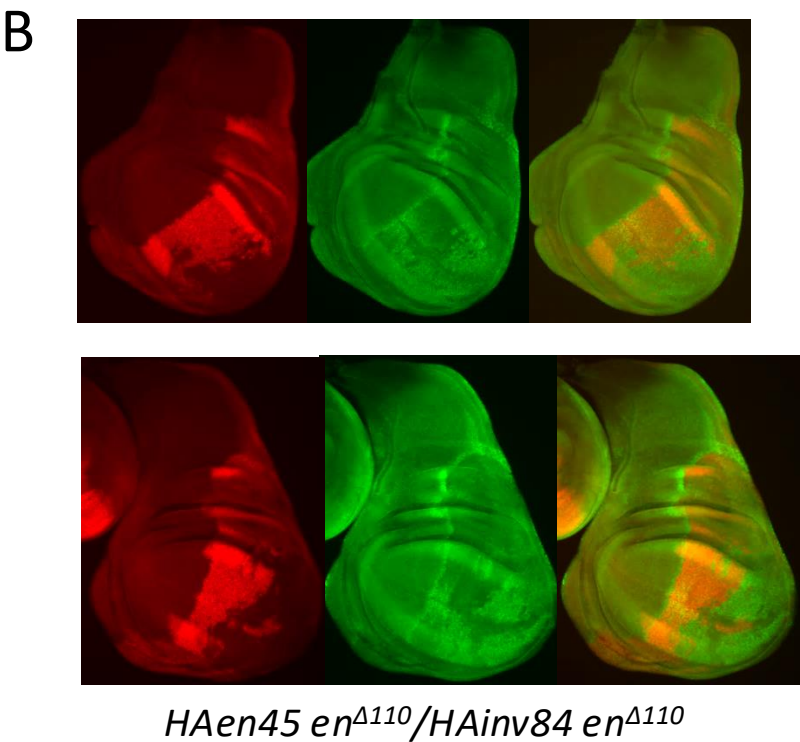

Supplemental Fig. 2

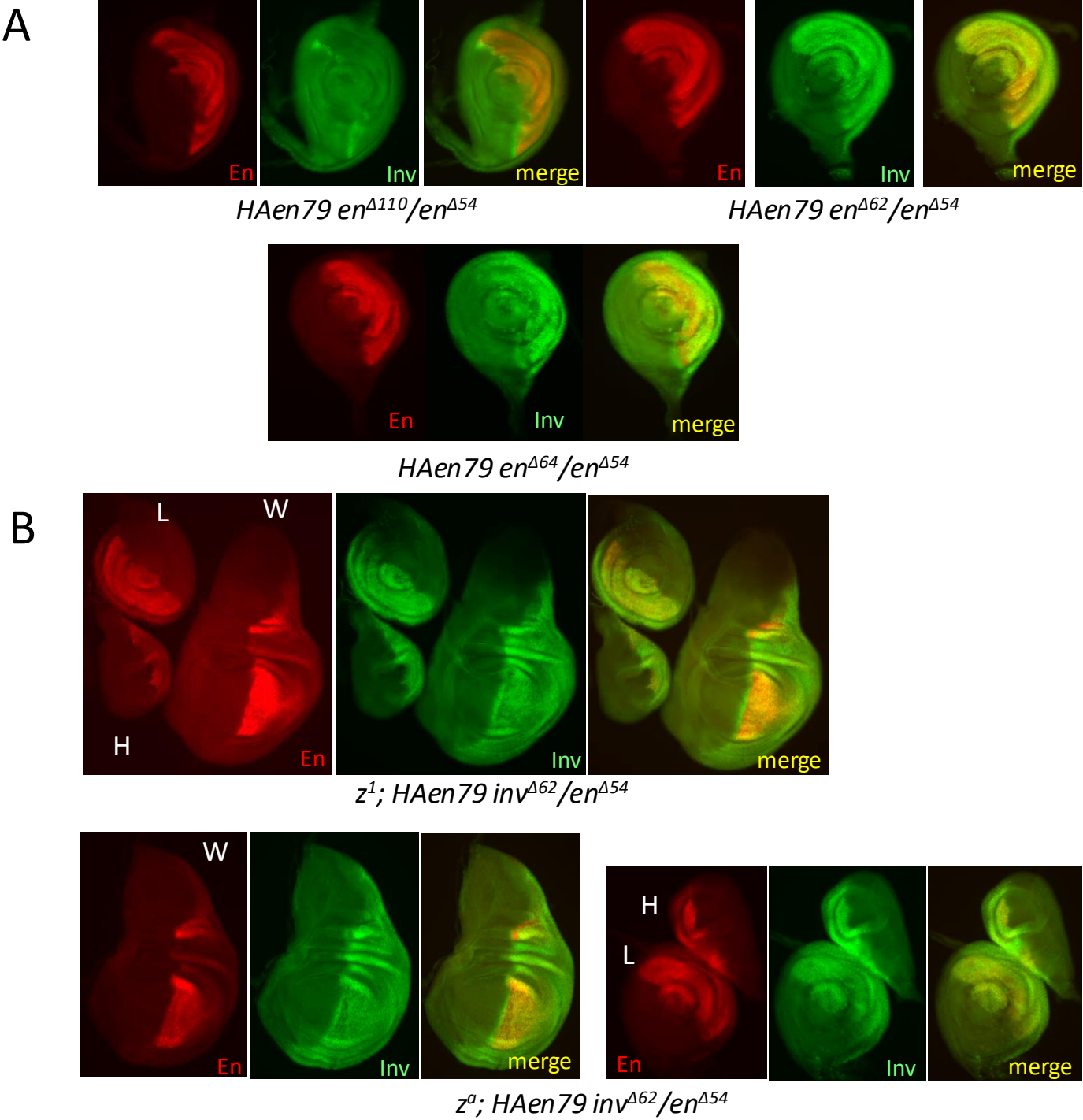

Supplemental Figure 3

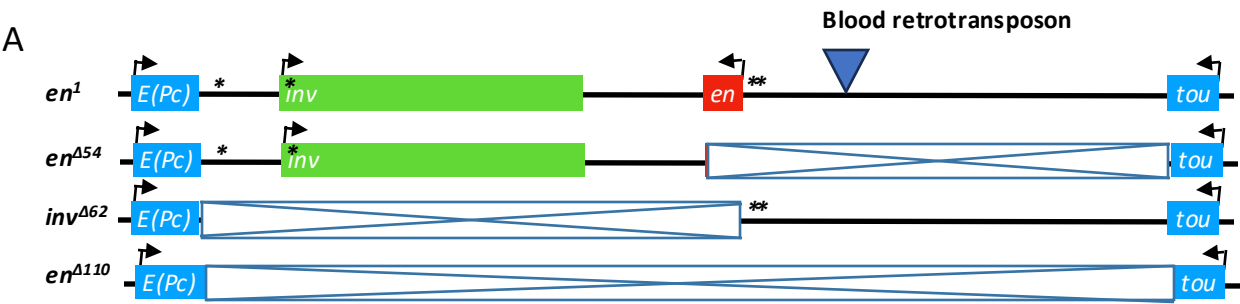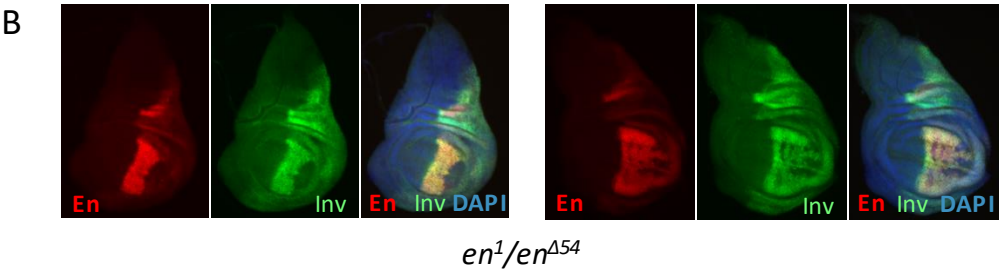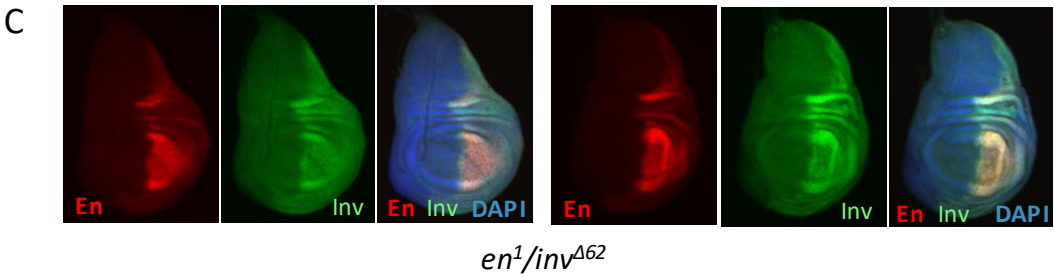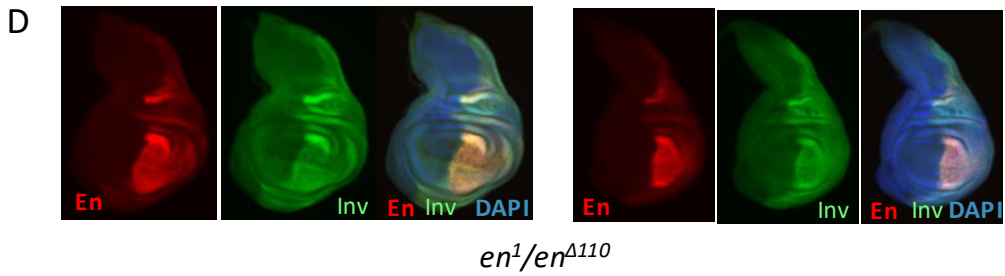

Supplement: iyaf276_Supplementary_Data [file iyaf276_supplementary_data.zip › Supplemental_Material_GENETICS-2025-308636.pdf]
